# Barriers and facilitative factors in the implementation of workplace health promotion activities in small and medium-sized enterprises: a qualitative study

**DOI:** 10.1186/s43058-022-00268-4

**Published:** 2022-03-02

**Authors:** Junko Saito, Miyuki Odawara, Hirokazu Takahashi, Maiko Fujimori, Akiko Yaguchi-Saito, Manami Inoue, Yosuke Uchitomi, Taichi Shimazu

**Affiliations:** 1 Division of Behavioral Sciences, National Cancer Center Institute for Cancer Control, 5-1-1 Tsukiji, Chuo-ku, Tokyo, 104-0045 Japan; 2 Division of Screening Assessment and Management, National Cancer Center Institute for Cancer Control, 5-1-1 Tsukiji, Chuo-ku, Tokyo, 104-0045 Japan; 3 Division of Supportive Care, Survivorship and Translational Research, National Cancer Center Institute for Cancer Control, 5-1-1 Tsukiji, Chuo-ku, Tokyo, 104-0045 Japan; 4 Division of Prevention, National Cancer Center Institute for Cancer Control, 5-1-1 Tsukiji, Chuo-ku, Tokyo, 104-0045 Japan; 5 Innovation Center for Supportive, Palliative and Psychosocial Care, National Cancer Center Hospital, Tokyo, Japan

**Keywords:** Facilitative factors, Barriers, Workplace health promotion, Implementation, Health manager, Employer, Small and medium-sized enterprises, Non-communicable diseases

## Abstract

**Background:**

There is an immense difference between large companies and small and medium-sized enterprises (SMEs) in implementation of evidence-based interventions (EBIs). Previous literature reveals various barriers that SMEs face during implementation, such as a lack of time, accessibility, and resources. However, few studies have comprehensively examined those influential factors at multi-levels. This study aims to identify the factors influencing the implementation of non-communicable disease prevention activities (tobacco, alcohol, diet, physical activity, and health check-up) in SMEs using Consolidated Framework for Implementation Research (CFIR).

**Methods:**

We conducted 15 semi-structured interviews with health managers and/or employers in 15 enterprises with less than 300 employees, and four focus groups among public health nurses/nutritionists of health insurers who support SMEs in four prefectures across Japan. A qualitative content analysis by a deductive directed approach was performed. After coding the interview transcript text into the CFIR framework constructs by two independent researchers, the coding results were compared and revised in each enterprise until an agreement was reached.

**Results:**

Of the 39 CFIR constructs, 25 were facilitative and 7 were inhibitory for workplace health promotion implementation in SMEs, which were across individual, internal, and external levels. In particular, the leadership engagement of employers in implementing the workplace health promotion activities was identified as a fundamental factor which may influence other facilitators, including “access to knowledge and information,” “relative priority,” “learning climate,” at organizational level, and “self-efficacy” at the health manager level. The main barrier was the beliefs held by the employer/manager that “health management is one’s own responsibility.”

**Conclusions:**

Multi-level factors influencing the implementation of non-communicable diseases prevention activities in SMEs were identified. In resource-poor settings, strong endorsement and support, and positive feedback from employers would be important for health managers and employees to be highly motivated and promote or participate in health promotion. Future studies are needed to develop context-specific strategies based on identified barriers and facilitative factors, and empirically evaluate them, which would contribute to narrowing the differences in worksite health promotion implementation by company size.

**Supplementary Information:**

The online version contains supplementary material available at 10.1186/s43058-022-00268-4.

Contributions to the literature

- There is little research to guide policymakers on how to narrow the differences in the implementation of evidence-based health promotion interventions between SMEs and large companies.
- Multi-level factors identified using a comprehensive framework of implementation research offer suggestions for context-specific strategies to increase the implementation of evidence-based interventions that address NCD prevention among SMEs.
- In particular, the leadership engagement of employers to implement the workplace health promotion activities was identified as a fundamental factor which may influence other facilitators; therefore, focusing on employers’ leadership engagement (commitment, involvement, and accountability) can be an effective strategy to improve implementation.

## Introduction

Non-communicable diseases (NCDs) are the leading causes of death and disability in working-age adults globally. Over 80% of all premature NCD deaths occur due to cardiovascular diseases, cancers, respiratory diseases, and diabetes [1]. The primary behavioral risk factors for death due to an NCDs are tobacco use, physical inactivity, harmful alcohol use, and an unhealthy diet [2]. Workplaces are good settings for adopting and implementing health promotion programs that address NCD prevention, owing to the high prevalence of risky health behaviors among the working-age population and the presence of infrastructure to offer such programs that have a wide reach over a longer duration [3, 4]. Several systematic reviews have revealed the effectiveness of workplace health promotion (WHP) interventions targeting dietary behaviors [5], tobacco use [6], and mental health [7], while reviews of interventions targeting physical inactivity and risky alcohol use have shown mixed results [8, 9].

The implementation of WHP interventions have massive differences between small and medium-sized enterprises (SMEs) and large companies, and this trend has persisted over the past 3 decades [10–12]. For example, in 2017, 39.5% of large US worksites with 500 or more employees offered all five elements of a comprehensive program (as defined by Healthy People 2010), whereas only 11.0% of small worksites with fewer than 25 employees offer these components [10, 11]. In Japan, the proportion of implementation between SMEs with less than 50 employees and large companies with more than 500 employees are 57.6% vs 99.1% for mental health measures and 12.9–14.5% vs 19.0–21.3% for complete smoke-free policies, respectively [13]. To promote WHP in SMEs with less than 50 employees, the Japanese government has established regional occupational health centers as public health facilities in 350 districts across Japan since 1993, but its utilization is limited [14]. Since 2015, the government also started the “Health and Productivity Management” approach to strategically promote employees’ health from a corporate management perspective, including a certification system for companies [15], but the number of certified companies is still very limited. A national survey showed that approximately only 20% of all SMEs are currently implementing any activities related to health and productivity management [16]. One of the main challenges that SMEs face during WHP implementation is that they do not know how to proceed with specific measures to combat their own health challenges [17], such as promoting healthy diet, providing support for smoking cessation, and consulting a doctor when recommended at medical check-ups.

Implementation strategies, one of the key concepts of implementation science, respond to the question of “how” to improve the adoption and integration of evidence-based health interventions into routine policies and practices within specific settings. If effective implementation strategies to promote WHP implementation are identified and provided in SMEs, it would reduce the difference in implementation between SMEs and large companies. However, the current evidence on the strategies for WHP implementation that target NCDs are sparse and inconsistent [18]. Theoretical implementation frameworks, such as the Consolidated Framework for Implementation Research (CFIR), suggest that factors influencing implementation may exist at the individual, organizational, cultural, or social level [19]. It is important to have a comprehensive understanding of the barriers and facilitators that influence the implementation process at SMEs, which can be used to identify the context-specific implementation strategies.

The evidence regarding barriers and facilitators that influence multi-level WHP implementation is quite limited, especially among SMEs in Asian countries. A recent review about the process evaluation for WHP identified that most of the barriers and/or facilitators in the USA and Europe were related to the inner setting of the enterprises including management support and lack of resources [20], and only two studies identified factors at social level beyond the enterprise (e.g., compatibility of program with societal developments, and competitive business environment) [21, 22]. Another review paper on health promotion in SMEs in the USA also revealed that the main barriers on WHP implementation were the inner setting of the enterprises, including few service providers, low commitment, and low internal capacity to implement the program [20, 23, 24]. However, most of these literatures were from the USA or Europe, and the evidence regarding barriers and facilitators that influence multi-level WHP implementation in Asian SMEs is quite limited [25–28]. Worksite contextual factors, including organizational culture, resources, and structures, and their relationships with WHP implementation may be different across regions and countries. A previous study suggested that organizational cultural factors were related to the effectiveness of organizations in North America, but not in Asian organizations including Japan [25–27, 29].

Thus, to reduce difference in WHP implementation between SMEs and large companies in Asian countries, this study aimed to identify barriers and facilitators at multiple levels beyond the inner setting for the implementation of WHP programs targeting NCD prevention among SMEs in Japan.

## Methods

In this qualitative study, two types of interviews were conducted to obtain the perspective of service providers: (1) 15 semi-structured interviews with persons in charge of health management at SMEs (health managers) and/or employers, and (2) four focus groups with public health nurses from the health insurance association/nutritionists, who support these SMEs. Because this study focused on the context of WHP implementation at SMEs, with high diverse WHP measures and contexts among them, semi-structured interviews were conducted for SMEs individually [30], whereas focus groups were conducted with public health nurses, who supporting different SMEs, to generate a rich understanding of their diverse experiences through interactions [30, 31], as the public health nurses in each branch of the Japan Health Insurance Association (JHIA) are pre-existing groups and active discussion was expected. The CFIR was adopted as a guide for the interviews, coding, and analysis. The targeted WHP activities were the following five NCD prevention measures—tobacco, alcohol, diet, physical activity, and health check-ups. The study protocol was approved by the Ethical Committee of the National Cancer Center Japan (No. 2019-034). Our report adheres to the standards for reporting qualitative research (SRQR) (supplementary file 1) [32].

### Sample selection and procedure

This study was conducted with the cooperation of the JHIA, the largest medical insurer in Japan covering approximately 2.4 million enterprises [33, 34]. Most of JHIA member enterprises are SMEs, and more than 90% of them have less than 30 employees [35].

The JHIA has 47 branches covering all prefectures across Japan, and each branch issues a certification of “health declaration” to enterprises that volunteer to actively work towards improving employee health. Over 60,000 enterprises have been certified with a “health declaration” as of 2021 [36]. Once certified, a health manager is appointed at each enterprise to plan and implement health promotion activities, with support from public health nurses affiliated with the association. In most cases, certification is offered on a continual rather than a renewal basis. In all SMEs except for one, administrative staff such as those in the general affairs department were assigned to be health managers and were allotted health management tasks in addition to their regular duties.

Two-stage purposeful sampling was used to recruit public health nurses and select enterprises. In the first stage, the central office of JHIA selected four branch offices that have experience in providing health promotion support at the organizational level. In the second stage, a leader or sub-leader of the public health division at each of the four branch offices selected three to five enterprises according to the following inclusion criteria: (1) qualify as a SME (100 or less employees in case of a service enterprise, 50 or less for retail, 100 or less for wholesale, and 300 or less for manufacturing and others) [37], (2) have already participated in the “health declaration” initiative, and (3) have already implemented activities for workplace health promotion. For criterion (1), if the enterprise is part of a branch of companies, the number of employees at the particular branch office was considered. Fifteen enterprises that matched the inclusion criteria were identified. We planned to recruit 20 SMEs and four focus groups at maximum to ensure theme saturation (i.e., no new themes were discovered through additional interviews [38]). During the analysis process, the core members of the study (JS, MO, and TS) discussed theme saturation, and consensus on data saturation was achieved upon the completion of 15 interviews and four focus groups, respectively.

For the semi-structured interviews, at least one health manager participated in the interviews from each enterprise, and the employer also participated in the same interview for each enterprise if they were available. The employer in this study is referred to as the chief executive officer. We invited employers and health managers in the same interview, because it is practical for them to discuss factors, strategies, and measures for WHP together in the context of real-world implementation. For the focus group, four to six public health nurses/nutritionists participated from each branch office. In total, eight employers and 22 health managers participated in the interviews and 20 public health nurses/nutritionists participated in the focus groups. In order to conduct the focus groups effectively, they were asked to respond to a one-page questionnaire in advance regarding the WHP activities being implemented at the enterprises they provided their services to. JS, MO, HT, and TS conducted the interviews and focus groups, and JS was trained in 2-day qualitative research training course. JS, MO, and TS are the implementation science researchers. All four interviewed researchers were not known to the participants of this research prior to conducting the study. For both semi-structured interviews and focus groups, we obtained verbal consent for participation from each participant prior to data collection.

### Measures

We developed an interview guide using the following five main domains based on CFIR: (i) intervention characteristics, (ii) outer setting, (iii) inner setting, (iv) individual characteristics, and (v) processes [19] (supplementary file 2). For both semi-structured interviews and focus groups, we focused on the specific topics and activities that the enterprises had agreed to implement at the time of adopting the health declaration. We asked open-ended questions focusing on the context (barriers and facilitators) within which the current activities were being implemented. For focus groups consisting of JHIA public health nurses, the emphasis was not on the support they provide for enterprises, but about their perceptions of what factors influenced the current activities among target enterprises. Instead of asking questions related to each sub-construct within the CFIR, we encouraged the interviewees to openly speak about each CFIR domain (e.g., what had been challenging or favorable with respect to adopting and implementing the current WHP activities), in order to gather information that they perceived to be important. We used probing questions only when the interviewee did not talk about a particular CFIR sub-construct. Each interview lasted approximately 60 min, while each focus group lasted approximately 120 min. Interviews and focus groups were conducted in Japanese, and audio-recorded, transcribed, and checked for accuracy.

### Data analysis

We qualitatively analyzed the data using a deductive approach [39]. The analysis, for both the interviews and focus groups, was performed in five steps. First, two out of three authors (JS, MO, AY-S) independently coded units of the transcript text according to the CFIR constructs. Second, the authors compared the coding results of the data for each enterprise and revised it until a consensus was reached. If there were units of transcript text that did not fit into any CFIR construct, a new construct was created inductively and coded. Third, a diagram depicting the relationships between the constructs was drawn for each enterprise in order to comprehensively understand the influential factors [40]. Using the coding results from both the interview and focus groups, either of the two authors (JS, MO) independently identified the relationships between constructs for each enterprise, and then discussed and revised it until they agreed on the final diagram. They further developed a summary memo, organized according to the CFIR constructs, for each enterprise. The summary memo was discussed with a third researcher (TS), who was not involved in the coding process, to achieve consensual validation. To further strengthen the credibility of the results, the preliminary summary memo with a description was shown to a few public health nurse participants to confirm that the views of health managers/employers were appropriately reflected. Finally, the barriers and facilitators, as per the CFIR constructs, were identified. The data from focus groups was also coded and categorized into CFIR sub-constructs and used to supplement the results of the employer and/or health managers’ interviews.

## Results

The enterprises included in this study conducted several WHP activities to prevent NCDs, except risky alcohol use prevention. Tables 1 and 2 show the characteristics of enterprise and participants. No enterprise included in this study comprised a branch of companies. Of the 15 enterprises, the data from one enterprise were treated as complementary data which was the same as the focus groups, because during the interview, it was found that they were a cooperative union and supported the health promotion activities at its member establishments, instead of conducting WHP activities for their own employees.

**Table 1:** Characteristics and workplace health promotion activities conducted in participating enterprises (*n* = 14)

Industry	
Construction	2
Manufacturing	3
Electricity, gas, heat supply, and water	1
Transport and postal services	2
Wholesale and retail trade	2
Scientific research, professional and technical services	3
Services, n.e.c.	1
Size	
< 50	6
50–99	6
≥ 100	2
Worksite health promotion activities	
Smoke-free policies	
Complete bans with reduction of smoking prevalence	4
Partial bans	3
Regular checks of blood pressure	
Regular monitoring of blood pressure	2
Only placing sphygmomanometer	1
Not yet placing sphygmomanometer	1
Diet	
Providing healthy menu at lunch time	1
Physical activity	
Physical activity	3
Health check-ups	
Encouraging participation in screening and lifestyle interventions conducted by insurers	3

One enterprise whose interview data were not treated as complementary data is not included in this table

**Table 2:** Characteristics of participants

	*n* (mean)	% (sd)
Semi-structured interviews (*n* = 15, participants *n* = 30)		
Sex		
Male	22	73.3
Female	8	26.7
Position title		
Employer (CEO)	8	26.7
Health manager		
Director	12	40.0
Section chief	4	13.3
Others	6	20.0
Focus groups (*n* = 4, participants *n* = 20)		
Sex		
Female	20	100.0
Job title		
Public health nurse	18	90.0
Nutritionist	2	10.0

CEO Chief executive officer

Of the 39 CFIR constructs assessed, 28 were facilitative and eight were inhibitory for WHP program implementation among SMEs (specifically, 25 were facilitative and eight were inhibitory from the semi-structured interviews and eight were facilitative and one were inhibitory from the focus groups) (Table 3). The factors identified from the focus groups were similar to the results from the semi-structured interviews and complemented the interview results. Three factors were identified as specific to focus groups: “Structural characteristics” in Inner setting domain, “Other personal attributes skills” in Individual characteristics domain (factors that are difficult to examine without the objective comparison of multiple companies) and “Champions” in the process domain (factors that are difficult to identify without objective observation from outside the company). Interventions not listed in the recommend programs as per CDC workplace Health Strategies were not included in the analysis (e.g., setting aside one day a month to not eat sweets, the full list of excluded interventions are shown in supplementary files 3). Quotes were labeled by the enterprise (alphabetically anonymized), the respondent (health manager or employer), and activity topics. Due to the word limit, selected results regarded as salient themes are shown in the manuscript. The full results with quotes, the constructs identified from the focus groups, and a table of factors and barriers by CFIR constructs according to each topic of the WHP activities are shown in supplementary files 4, 5 and 6.

**Table 3:** Barriers and facilitators in workplace health promotion activity implementation according to CFIR constructs

CFIR Domain and constructs	Facilitators	Barriers
I. Intervention characteristics		
A. Intervention source	・Voluntary motivation to promote employees’ health and externally provided referral which was matched their needs [Interviews]	
B. Evidence strength and quality	・The employer’s perception of the effectiveness of the activities [Interviews]	
C. Relative advantage	・Recognition of its relative advantages over other topics within NCD prevention [Interviews]	
F. Complexities	・Recognition of its easiness to adopt [Interviews] [Focus groups]	
II. Outer setting		
A. Needs and resources of those served by the organization	・Embedded system to understand employees’ needs [Interviews]	・Resistance of smokers in case of tobacco control activities [Interviews] [Focus groups]
B. Cosmopolitanism	・Networking with other companies [Interviews]	
C. Peer pressure	・Existence of companies in a competitive relationship [Interviews] [Focus groups]	
D. External policy and incentives	・Legal obligation, the certification of WHP activities [Interviews]	
III. Inner setting		
A. Structural characteristics	・The smaller company size [Focus groups]	
B. Networks and communications	・Formal (e.g., health committees) and/or unformal communication (e.g., feedback shared during daily communication) [Interviews] [Focus groups] ・Relationships of mutual trust between the employer and the employees [Interviews]	
C. Culture	・ Family like culture in the workplace [Interviews]	
D. Implementation climate		
1. Tension for change	・The sense of urgency for change regarding health promotion of employees [Interviews]	・A lack of motivation or sense of urgency to implement WHP activities now [Interviews]
2. Compatibility	・Alignment with employees’ business processes [Interviews]	・The time to spend the WHP activities [Interviews]
3. Relative priority	・Higher prioritization of WHP activities [Interviews]	・ Lower priority compared to customer-focused activities or productivity [Interviews]
4. Organizational incentives and rewards	・Praising the participants of WHP activities in a meeting [Interviews]	
5. Goals and feedback	・Creation of a common understanding of the goals and objectives for WHP activities among all employees [Interviews]	
6. Learning climate	・The employer’s perception that the health manager(s) is an indispensable and knowledgeable person in the WHP implementation [Interviews]	
E. Readiness for implementation		
1. Leadership engagement	・Communicating the company's philosophy linked to the WHP to all the employees [Interviews] [Focus groups] ・Supporting those who are engaging the implementation [Interviews]	・Limited provision of resources and support from the employer [Interviews]
3. Access to knowledge and information	・Access to external knowledge and information, such as participation in study sessions and support from JAIH health nurses [Interviews]	・Limited access to external knowledge and information made it difficult [Interviews]
IV. Characteristics of individuals		
A. Knowledge and beliefs about the innovation	・Awareness to conduct WHP activities as part of their regular task [Interviews]	・Employer or health manager’s beliefs that people need to take full responsibility for their own health [Interviews]
B. Self-efficacy	・Health manager’s self-efficacy as a result of the employer's entrusting him/her with health promotion [Interviews]	
D. Individual identification with organization	・Relationships of mutual trust between the employer and the employees [Interviews]	
E. Other personal attributes	・Health manager’s skills and authority [Focus groups]	
V. Process		
1. Opinion leaders	・Involvement of a person who has a strong influential power in the company [Interviews]	
2. Formally appointed internal implementation leaders	・Existence of health manager formally appointed by the employer to implement WHP activities [Interviews]	・Absence of health manager formally appointed by the employer to implement WHP activities [Interviews]
3. Champions	・Involving front-line champions [Focus groups]	
4. External change agents	・Support from public health nurses or nutritionists at JHIA [Interviews]	
D. Reflecting and evaluating	・Personal reflection by health managers using annual health report by JHIA [Interviews]	

*CFIR* Consolidated Framework for Implementation Research, *JHIA* Japan Health Insurance Association, *NCD* Non-communicable disease, *WHP* worksite health promotion

### Intervention characteristic domain

#### Relative advantage

When deciding on a topic for the WHP activity, when the health manager recognized its relative advantages over other topics within NCD prevention, it was more likely to be selected and be proactively implemented. At one enterprise, the health manager selected physical activity since it is relevant for all ages and allows everyone to participate in and benefit from it, as compared to other interventions such as those related to smoking or blood pressure.

### Outer setting domain

#### Cosmopolitanism

One health manager mentioned the advantage of networking with other companies on program implementation. In the case of company located within an industrial park sharing the health check-up bus, the implementation of health check-up was perceived to be highly advantageous in terms of leading to a collaboration with other organizations in the industrial park.

Now, all of the employees in this industrial park gather (to receive health check-ups). Until four or five years ago, only our company had done them. (D, health manager, health check-ups)

The health manager of the cooperative union (the enterprise recruited as an interview target, and later treated as complementary data same as focus groups of public health nurses) reported that it was effective to make an opportunity for health managers from various companies to meet each other and share their concerns and ideas, as most of them were conducting WHP activities by themselves.

### Inner setting domain

#### Relative priority

Many health managers mentioned that the enterprise’s prioritization of WHP activities was relative to other things as a facilitative factor. Specifically, if health management was a part of the company’s overall management vision, it was easy to obtain the leader’s approval and implement health promotion measures immediately.

It’s going to cost, and we talked to the employer and (health and safety) committee. [ … ] The most important thing was that it would help employees manage their health. So, we got the go-ahead right away. (F, health manager, tobacco control)

However, one employer mentioned that WHP implementation was a lower priority compared to customer-focused activities or productivity. Such a relatively low priority can be a barrier to implementation and is likely to be highly dependent on the business conditions of SMEs at any given time.

#### Learning climate

When health managers feel that the employer perceived them as an indispensable and knowledgeable person in the WHP implementation, they proactively examine, plan, and implement the WHP activities. In one enterprise, the health manager, who previously had no knowledge of health management, but was trusted by the employer and assigned this task, proactively implemented the program through trial and error. When the implementation went well, the manager felt affirmed, raising their “self-efficacy”, and the motivation to continue the program, and the implementation of other activities further increased, thereby, creating a virtuous cycle.

The representative just told me he wanted to do health management for the employees. It was a great learning experience for me to work on our own." "(When deciding on the WHP activities to adopt) The employer basically gave me permission to select whichever I wanted. [ … ] I didn’t ask (my superiors) which one they preferred. We kind of just said, ‘This is the one we’ll go with (A, health manager, physical activity)

#### Leadership engagement

There were two ways in which employers engaged in WHP activities—communicating the company’s philosophy linked to the WHP to all the employees, and supporting those who are engaging the implementation—both of which were strong drivers of implementation. Direct and repeated communication from the employer at general meetings and other occasions led others within the company to relatively prioritize WHP activities more and, hence, implementation progressed.

The current representative of the company believes that the happiness of employees and those close to them will lead to contributions to customers and the local community. [ … ] I think the most important thing is the representative’s way of thinking. (A, health manager, physical activity)

Similarly, extending support to those in charge of the program, such as allowing them to participate in external trainings related to WHP program implementation during working hours, facilitated implementation.

I was told that I can participate in such things (such as seminars on WHPs outside the company) as much as I want because they see it as part of my work. (A, health manager, physical activity)

Multiple public health nurses/nutritionists in the focus groups supported these findings, as they also mentioned that “The employer’s voice is essential,” and “The influence of employers and health care managers is significant in ensuring the sustainability of WHP implementation.”

On the other hand, health managers who were not given enough time or support to implement WHP-related tasks inevitably gave lower priority for WHP implementation. In this enterprise, 1 year after declaring that they would perform blood pressure control activities, they still had not purchased a blood pressure monitor.

I’d like to help where I can (for implementing WHP activities), but I’m so busy with my other duties and I tend to forget. (C, health manager, blood pressure)

#### Access to knowledge and information

As many SMEs did not have existing resources to initiate WHP activities, many health managers reported that access to external knowledge and information, such as participation in study sessions during working hours and support from JHIA health nurses, was necessary to proceed the implementation. This accessibility to information was enhanced by support from the employer and the positive attitudes of health managers.

In contrast, when access to such external knowledge and information was difficult, even if the sense of urgency in the health manager increased, it did not lead to the actual implementation. In one enterprise implementing blood pressure management, nothing was implemented after installing blood pressure monitors despite having a sense of urgency to do something more, because they did not know what to do and had poor access to knowledge and information.

There are many employees with high blood pressure, so we need to think of something, but I’m not sure what I can do at work. (B, health, manager; blood pressure)

### Characteristics of individuals

#### Knowledge and beliefs about the intervention

Some employers and health managers reported that they were clearly aware of having to conduct WHP activities as part of their regular task, rather than as an additional task, as they believed that the health promotion of employees is one of the issues the enterprise should engage in.

Employees are the most important. In order to keep employees to work with high motivation for a long time, (spending resources) for their well-being is an investment, not a cost. (K, employer, tobacco control and health check-ups)

However, some employers or health managers were convinced that health behavior would not change unless each employee’s awareness is changed first, and it led to the belief that the WHP activities would have a limited effect, as a result of which the actual implementation was limited.

It’s not good if the person themselves is not aware of what’s going on. [ … ] I try to do things for myself. [ … ] I’m diabetic, so I’m trying hard to lower my blood pressure, but until each of us is aware of it, it won’t affect us (no matter what those around us say). (B, health manager, blood pressure)

#### Self-efficacy

Some employers reported, or health manager reported as an employer’s perception, that they (employers) entrusted health managers with the task of health promotion and they were able to accomplish it with the help of adequate time and manpower. Then, the managers’ sense of self-efficacy increased, thereby leading to a virtuous cycle and continued implementation in the subsequent years (see “learning climate” and “peer pressure” as well).

#### Individual identification with organization

Some health managers described that the employer’s sincere concern for the employees lead the employees’ desire to respond to the employer’s concern for them, and such relationships of mutual trust between the employer and the employees facilitated implementation.

#### Other personal attributes

In the focus groups, the public health nurses suggested that the health manager’s skills and authority influenced the implementation directly. Although it was not necessarily related to their position, it was important for health managers to have the authority to speak in such a way that employees would listen to them, especially with respect to the continuity of WHP activities.

### Process domain

#### Change agent

Most of employers and health managers perceived the public health nurses or nutritionists at JHIA as key members when implementing WHP activities, as they provided useful advice or information about WHP. In addition, they perceived that health lectures by public health nurses are more effective as employees were more receptive to the information coming from them.

#### Champions

The public health nurses further suggested that involving front-line champions advanced program implementation. For example, when adopting measures to make the company cafeteria menu healthier, discussing the issue among all stakeholders, that is, not only the employer and general manager, but also the cook, led to the program’s successful adoption and implementation.

## Discussion

We evaluated the implementation of health promotion activities in SMEs using a qualitative approach guided by CFIR and identified constructs across five domains that facilitated and inhibited implementation.

### Leadership engagement of employers as a fundamental factor

The diagram depicting the relationships between CFIR constructs showed that the “leadership engagement” of employers to implement the WHP activities influenced other facilitators. Employers’ “leadership engagement” in this study refers to the commitment, involvement, and accountability of employers with regard to the implementation of WHP, with a sincere belief that they value the health and well-being of their employees [19]. The leadership engagement of employers may increase the “access to knowledge and information” of health managers and foster the “implementation climate” which refers to the targeted employees’ shared perceptions of the extent to which the use of a specific innovation is “rewarded, supported, and expected within their organization” [41]. Those two factors (i.e., increased “knowledge and information” and improved “implementation climate”) caused among health managers greater motivation and skills, and “self-efficacy” regarding the implementation of WHP activities.

Our findings supported a theory of the organizational determinants of WHP implementation, which showed that organizations can strengthen the implementation climate by facilitating knowledge and skills development in employees [42]. To create an “implementation climate” in SMEs, it would be important to first get the buy-in and leadership of employers to set up the resources and systems for WHP [43]. This would allow health managers, who are often the only front-line implementers in the organization, to increase their knowledge and skills in implementing WHPs, which would strengthen their implementation climate, which would then be shared with employees and become a company-wide climate.

The leadership engagement of employers is a common key factor of the best practices of WHP programs [44, 45], and previous empirical studies showed that leadership support is associated with implementation processes and work attendance [46]. Our study further suggested possible mechanisms by showing several factors that may link the relationships between leadership engagement and improved implementation of WHPs. The leadership engagement is considered to impact WHP programs by creating a culture of health [43], and the factors that may link the relationships identified in this study may also contribute to creating such a culture of health. Especially, in small-sized companies, where organizational layers are fewer than in larger companies, it is easier for the employers’ beliefs and vision to permeate the entire company [47].

The effects of leadership engagement must take into consideration the culture in which they perform [48]. Compared to other countries, Japan has moderate power distance and collectivism, such as importance for values regarding social obligation, social harmony, and social contribution [49–51]. We found that employers’ sincere desire for employees’ well-being, trust, and acclaim for employees and health managers will led to the employees’ trust in the employers, which in turn can also facilitate implementation. When management involves employees in various ways, employees would be encouraged to have more positive attitudes towards not only the employer and their own self, but also towards the organization [52]. Especially in Japan, with collectivism culture, transformational leadership is more likely to empower employees than charismatic leadership [53], and those reciprocal relationships between employees and the employer and/or organization may be easily conceived.

### Barriers in implementing WHP activities

Beliefs held by the employer and/or manager that “health management is one’s own responsibility” was suggested to be a barrier for implementation. When employers and managers feel strongly that the promotion of employees ultimately depends on individual employees’ mindset regardless of what the company does, they are less likely to fully utilize the resources for such programs. In other words, a lack of belief about the effectiveness of health promotion at the workplace inhibited strong support and proactive implementation of WHPs. In the management system of Japanese companies, decisions are generally taken solely by the employer in SMEs, while large companies tend to make decisions by the consensus of the senior management [54]. In addition, those in charge of WHP implementation in SMEs are often a single health manager (e.g., a staff member from departments of human resources or general affairs), while they are a team in large companies. Therefore, the perceptions of the individual employer and/or manager may be more likely to directly affect the implementation of WHP.

Limited resources (people, goods, and money) to use for health promotion was not identified as a barrier in our study, despite it being one of the main barriers for SMEs in implementing WHP [24]. Such inconsistent findings with previous literature may be mainly because there was already a certain level of readiness for WHP implementation among target enterprises, as one of the inclusion criteria was that they had to have already made a health declaration; the implemented WHPs were activities with low initial investment, rather than packaged comprehensive programs; and fewer resources were needed as many of the establishments had fewer than 100 employees.

### Cosmopolitanism as a potential facilitator

One of the factors that may be unique to SMEs in this study was “cosmopolitanism.” The existing network between companies located in the same industrial park was effectively utilized for information exchange and resource sharing, and they facilitated implementation. In health care settings, collaborative learning across agencies is known to facilitate implementation of evidence-based interventions by altering social networks among participants to promote the transmission of new ideas and social support [55]. WHO also recommended “Partner and build alliances” as one of the five required actions for implementing health promotion and suggested priorities for the alliance action [56]. However, in the workplace, such a network between companies has not been focused on as an influential factor on WHP implementation, which maybe partly due to most of the evidence being obtained from larger companies where information and support are sufficient. Employers of SMEs often have regular gatherings with other business organizations or subcontractors within the same industries, and there is often a system in place to exchange opinions and information regarding business between them.

### Implications

The study findings implied that continuous support for both employers to encourage the leadership engagement and health managers to increases their knowledge, change their beliefs, and raise their self-efficacy may promote evidence-based WHP implementation. In most large companies, occupational health professionals are employed and stationed at the company to provide support for both WHP implementation at the organizational level and health behavior change at the individual level. However, in many SMEs, public health nurses of JHIA mainly support health behavior changes among high-risk employees as they have limited time to support each SME. To reduce differences in health behaviors among employees between SMEs and large companies, measures to tackle the social context at organizational level (i.e., implementation of WHP) are required [57]. Our findings reveal the importance of approaches to factors outside of workplaces in addition to those inside them, such as encouraging knowledge, beliefs, and self-efficacy of employers and health managers at the individual level, and supporting collaborations with other business organizations to accumulate knowledge on beset practices and shared learning related to WHP activities (*cosmopolitanism*). Thus, shifting the main target of limited resources of occupational health professionals from high-risk individuals to employers and health managers, as well as external factors that support internal WHP implementation would be a more efficient and sustainable way to support WHP implementation in SMEs.

### Strengths and limitations

To the best of our knowledge, this is the first study examining the barriers and facilitators for the implementation of WHP activities in SMEs using CFIR. Our findings will offer suggestions to develop implementation strategies for promoting WHP activities at SMEs in the future. The approach to categorizing the identified influential factors into CFIR constructs will promote the integration of findings with other implementation research using CFIR, and contribute to an understanding of the applicability of health-related interventions in various settings through a comparison of consistent and inconsistent findings. However, there are a number of limitations to this study that must be addressed. First, we collected data only from the providers (employer, health manager, and public health nurses), but not from those receiving the interventions (employees). Especially for the assessment of “patient’s needs” and “relative priority,” even if the provider states that the employees’ needs are understood or that the employers’ desire to prioritize and value health promotion at the workplace is conveyed to the employees, it may not necessarily reflect the truth unless we also ask the employees. Second, the generalizability of our findings may be limited as our sample enterprises had already participated in the “health declaration” initiative and implemented activities for workplace health promotion, which means the readiness for WHP implementation is high. In particular, for SMEs that have not implemented any activities related to health and productivity management in Japan (which is reported to be 80% of SMEs [16]), future studies are needed to identify the factors that inhibit WHP adoption and identify implementation strategies to overcome those barriers. Third, we could not evaluate the degree of influence quantitatively as it was difficult to compare five very different topics of the WHP activities with a wide range of intervention characteristics.

## Conclusions

Multi-level factors influencing the implementation of NCD prevention measures in SMEs were identified. Especially, leadership engagement by employers was identified as the most influential and fundamental factor. These findings highlight the need to focus on the internal and external structures of an enterprise. In resource-poor settings, strong endorsement and support, and positive feedback from employers was important for health managers and employees to be highly motivated to promote or participate in health promotion; this led to the continuous implementation of or participation in health promotion activities, thus creating a positive cycle. We recommend the development of future health promotion programs at SMEs using strategies that enhance these multi-level facilitative factors.

## Supplementary Information

**Additional file 1: Supplementary file 1.** SRQR guidelines for reporting qualitative research studies.

**Additional file 2: Supplementary file 2.** Interview guide for semi-structured interviews for employers and health managers.

**Additional file 3: Supplementary file 3.** List of interventions implemented in the participated SMEs that were not included in the analysis due to unknown evidence at worksite.

**Additional file 4: Supplementary file 4.** Full version of the results.

**Additional file 5: Supplementary file 5.** Summary of the findings from the focus groups.

**Additional file 6: Supplementary file 6.** Barriers and facilitators in workplace health promotion according to topics of activities.

## Data Availability

An anonymous analyzed data will be available to researchers upon reasonable request to the corresponding author.
