# Supplementary material for: Barriers and facilitative factors in the implementation of workplace health promotion activities in small and medium-sized enterprises: a qualitative study
Source: Implement Sci Commun. 2022 Mar 2;3:23. doi: 10.1186/s43058-022-00268-4 (PMC8889638; doi:10.1186/s43058-022-00268-4)
Supplement: Supplementary file 2 — Additional file 2: Supplementary file 2. Interview guide for semi-structured interviews for employers and health managers. [file 43058_2022_268_MOESM2_ESM.docx]

**Interview guide for semi-structured interviews for employers and health managers**

**Question 1: Basic information about you and your company**

First of all, could you give us an overview of your company?

Business type: ＿＿＿＿＿＿

Founding spirit: ＿＿＿＿＿＿

Management philosophy: ＿＿＿＿＿＿

Business description: ＿＿＿＿＿＿

Founding date: ＿＿＿＿＿＿

Number of employees: ＿＿＿＿＿＿

Health declaration certified date: ＿＿＿＿＿＿

Can you tell us about your department and your job role in your company?

What specific tasks are you responsible for in terms of health management?

**＿＿＿＿＿＿＿＿＿＿＿＿＿＿＿＿＿＿＿＿＿＿＿＿＿＿＿＿＿＿＿＿＿＿＿＿＿＿＿＿＿＿＿＿＿＿＿＿＿＿＿＿＿＿＿＿＿＿＿＿＿＿＿＿＿＿＿＿＿＿＿＿＿＿＿＿＿＿＿＿**

**Question 2: We would like to ask about specific health promotion measures in your company*. (Introductory question)***

**Can you tell us about any health issues (employee health problems) that you are aware of in your company?**

- What do you think are the causes or background of these health issues?

*(probing question)*

＿＿＿＿＿＿＿＿＿＿＿＿＿＿＿＿＿＿＿＿＿＿＿＿＿＿＿＿＿＿＿＿＿＿＿＿＿＿＿＿＿＿＿＿＿＿＿＿＿＿＿＿＿＿＿＿＿＿＿＿＿＿＿＿＿＿＿＿＿＿＿＿＿＿＿＿＿＿＿＿＿＿＿＿＿＿＿＿＿＿＿＿＿＿＿＿＿＿＿＿＿＿＿＿＿＿＿＿＿＿＿＿＿＿＿＿＿＿＿＿

**We would like to ask you about the specific measures your company is taking to tackle health issues in the "Health Declaration."**

Measures (1)

Date of registration of the declaration of a healthy workplace: ______________

Start of measures: ______________

Priority issues: ______________

Objectives: ______________

Health Plan: ______________

　Specific activities: ______________

Evaluation indicators: ______________

Ask the same questions for other measures, if there are other declared and implemented measures in health declaration.

**I would like to inquire a little more about each of the measures (1), (2), and (3) in chronological order. *(Transition statement)***

**In your company, who proposed the health issues to be addressed and through what process were they decided?**

- What was the relationship between the process above and its subsequent implementation? *(probing question)*

＿＿＿＿＿＿＿＿＿＿＿＿＿＿＿＿＿＿＿＿＿＿＿＿＿＿＿＿＿＿＿＿＿＿＿＿＿＿＿＿＿＿＿＿＿＿＿＿＿＿＿＿＿＿＿＿＿＿＿＿＿＿＿＿＿＿＿＿＿＿＿＿＿＿＿＿＿＿＿＿＿＿＿＿＿＿＿＿＿＿＿＿＿＿＿＿＿＿＿＿＿＿＿＿＿＿＿＿＿＿＿＿＿＿＿＿＿＿＿＿

**What was the decisive factor in choosing health issues to be addressed and measures to be adopted for your company?**

-What information was important to you and the CEO? What information were you and CEO looking for?

　　 -Advantages and disadvantages compared to other health measures

-The cost of implementing the measure

-The availability of procedures, tools, resources, etc. for implementation

(Were these deciding factors of your choice?)

*(probing question)*

**Question 3: Factors that inhibit or promote implementation *(Focus question)***

**What do you think is the most important thing for your company to implement these measures?**

＿＿＿＿＿＿＿＿＿＿＿＿＿＿＿＿＿＿＿＿＿＿＿＿＿＿＿＿＿＿＿＿＿＿＿＿＿＿＿＿＿＿＿＿＿＿＿＿＿＿＿＿＿＿＿＿＿＿＿＿＿＿＿＿＿＿＿＿＿＿＿＿＿＿＿＿＿＿＿＿＿＿＿＿＿＿＿＿＿＿＿＿＿＿＿＿＿＿＿＿＿＿＿＿＿＿＿＿＿＿＿＿＿＿＿＿＿＿＿＿

-What do you think is the most important thing required or that will encourage (promote) or have a positive impact on the implementation?

-What do you think are the most important things to do?

-Please tell us about any innovations that were effective in promoting the measures

at your company. *(probing statement)*

＿＿＿＿＿＿＿＿＿＿＿＿＿＿＿＿＿＿＿＿＿＿＿＿＿＿＿＿＿＿＿＿＿＿＿＿＿＿＿＿＿＿＿＿＿＿＿＿＿＿＿＿＿＿＿＿＿＿＿＿＿＿＿＿＿＿＿＿＿＿＿＿＿＿＿＿＿＿＿＿＿＿＿＿＿＿＿＿＿＿＿＿＿＿＿＿＿＿＿＿＿＿＿＿＿＿＿＿＿＿＿＿＿＿＿＿＿＿＿＿

-What do you think are the things that inhibit or negatively affect implementation?

-If you have any specific examples, please let us know. *(probing statement)*

＿＿＿＿＿＿＿＿＿＿＿＿＿＿＿＿＿＿＿＿＿＿＿＿＿＿＿＿＿＿＿＿＿＿＿＿＿＿＿＿＿＿＿＿＿＿＿＿＿＿＿＿＿＿＿＿＿＿＿＿＿＿＿＿＿＿＿＿＿＿＿＿＿＿＿＿＿＿＿＿＿＿＿＿＿＿＿＿＿＿＿＿＿＿＿＿＿＿＿＿＿＿＿＿＿＿＿＿＿＿＿＿＿＿＿＿＿＿＿＿

-What do you think would have the greatest impact on implementation in your company?

*(probing question)*

＿＿＿＿＿＿＿＿＿＿＿＿＿＿＿＿＿＿＿＿＿＿＿＿＿＿＿＿＿＿＿＿＿＿＿＿＿＿＿＿＿＿＿＿＿＿＿＿＿＿＿＿＿＿＿＿＿＿＿＿＿＿＿＿＿＿＿＿＿＿＿＿＿＿＿＿＿＿＿＿＿＿＿＿＿＿＿＿＿＿＿＿＿＿＿＿＿＿＿＿＿＿＿＿＿＿＿＿＿＿＿＿＿＿＿＿＿＿＿

**Question 4: Outer setting (*focusing on constructs not responded to in question 3*)**

**We would like to continue to ask you about things that you think have influenced your company to implement the measures.**

**Are there any other things or objects outside your company that you would like to mention?**

-For example, how did you feel about the following factors?

　 -the existence of other sites that have implemented measures or other similar programs

　 -the existence of county, national, or related organizations' evaluation or recognition

programs

-the impact of laws, guidelines

-the extent to which employees and executives interact with people and organizations outside the company, or participate in meetings, events, etc.

*(probing questions)*

＿＿＿＿＿＿＿＿＿＿＿＿＿＿＿＿＿＿＿＿＿＿＿＿＿＿＿＿＿＿＿＿＿＿＿＿＿＿＿＿＿＿＿＿＿＿＿＿＿＿＿＿＿＿＿＿＿＿＿＿＿＿＿＿＿＿＿＿＿＿＿＿＿＿＿＿＿＿＿＿＿＿＿＿＿＿＿＿＿＿＿＿＿＿＿＿＿＿＿＿＿＿＿＿＿＿＿＿＿＿＿＿＿＿＿＿＿＿＿＿

**Question 5: Inner setting (*focusing on constructs not responded to in question 3*)**

**Are there any other internal things in your company that you would like to mention?**

-For example, how did you feel about the following factors?

　 -The degree of daily communication within the company

　 -The degree to which the needs of your employees match the content of the measures

　 -The priority of the measures within the company

　 -The level of involvement of employers, executives, managers, etc.

-Company culture

　 -Resources the company currently have

*(probing questions)*

＿＿＿＿＿＿＿＿＿＿＿＿＿＿＿＿＿＿＿＿＿＿＿＿＿＿＿＿＿＿＿＿＿＿＿＿＿＿＿＿＿＿＿＿＿＿＿＿＿＿＿＿＿＿＿＿＿＿＿＿＿＿＿＿＿＿＿＿＿＿＿＿＿＿＿＿＿＿＿＿＿＿＿＿＿＿＿＿＿＿＿＿＿＿＿＿＿＿＿＿＿＿＿＿＿＿＿＿＿＿＿＿＿＿＿＿＿＿＿＿

**Question 6: Implementation process (*focusing on constructs not responded to in question 3*)**

**How was your company's process for setting goals, formulating specific plans, and conducting evaluations for implementing measures?**

＿＿＿＿＿＿＿＿＿＿＿＿＿＿＿＿＿＿＿＿＿＿＿＿＿＿＿＿＿＿＿＿＿＿＿＿＿＿＿＿＿＿＿＿＿＿＿＿＿＿＿＿＿＿＿＿＿＿＿＿＿＿＿＿＿＿＿＿＿＿＿＿＿＿＿＿＿＿＿＿＿＿＿＿＿＿＿＿＿＿＿＿＿＿＿＿＿＿＿＿＿＿＿＿＿＿＿＿＿＿＿＿＿＿＿＿＿＿＿＿

**Also, could you please tell us about the existence of internal and external stakeholders who have an influence on the implementation of measures in your company?**

＿＿＿＿＿＿＿＿＿＿＿＿＿＿＿＿＿＿＿＿＿＿＿＿＿＿＿＿＿＿＿＿＿＿＿＿＿＿＿＿＿＿＿＿＿＿＿＿＿＿＿＿＿＿＿＿＿＿＿＿＿＿＿＿＿＿＿＿＿＿＿＿＿＿＿＿＿＿＿＿＿＿＿＿＿＿＿＿＿＿＿＿＿＿＿＿＿＿＿＿＿＿＿＿＿＿＿＿＿＿＿＿＿＿＿＿＿＿＿＿

**Question 7: Individual characteristics (*focusing on constructs not responded to in question 3*)**

**We would like to ask you a few questions**

　　 -How much did you know initially about the effectiveness and specifics of these measures?

How about now? Did you receive adequate support? Specifically?

-How much did you believe in the effectiveness of these measures? Do you believe in them at present?

　 -How confident were you initially that you could implement these measures as the person

in charge? How confident are you now?

　　 -What resources or support do you think would increase your confidence?

　　 -How well do you communicate with the president, other managers, or employees about

these measures?

＿＿＿＿＿＿＿＿＿＿＿＿＿＿＿＿＿＿＿＿＿＿＿＿＿＿＿＿＿＿＿＿＿＿＿＿＿＿＿＿＿＿＿＿＿＿＿＿＿＿＿＿＿＿＿＿＿＿＿＿＿＿＿＿＿＿＿＿＿＿＿＿＿＿＿＿＿＿＿＿＿＿＿＿＿＿＿＿＿＿＿＿＿＿＿＿＿＿＿＿＿＿＿＿＿＿＿＿＿＿＿＿＿＿＿＿＿＿＿＿

**Question 8: Summary *(summary question)***

**What do you think the government, administrative agencies, or occupational health professionals should do to promote specific health guidance, examinations, and treatment at your company, and to further promote the implementation of health issues at your workplace?**

-What do you think the government, administrative agencies, or occupational health professionals should provide you to further promote the implementation of the health promotion measures?

-What kind of health plan is your company looking for?

＿＿＿＿＿＿＿＿＿＿＿＿＿＿＿＿＿＿＿＿＿＿＿＿＿＿＿＿＿＿＿＿＿＿＿＿＿＿＿＿＿＿＿＿＿＿＿＿＿＿＿＿＿＿＿＿＿＿＿＿＿＿＿＿＿＿＿＿＿＿＿＿＿＿＿＿＿＿＿＿＿＿＿＿＿＿＿＿＿＿＿＿＿＿＿＿＿＿＿＿＿＿＿＿＿＿＿＿＿＿＿＿＿＿＿＿＿＿＿＿

**As we are running out of time, we would like to summarize today's interview.**

**The following were mentioned as important things to promote specific health guidance, examination, and treatment, as well as in implementing health issues, especially ＿＿＿＿＿. Among them, ＿＿＿＿＿＿＿＿＿＿＿ was said to be important. We also received comments on ＿＿＿＿＿＿＿＿＿＿＿.**

**Do you have any comments on these summaries?**

＿＿＿＿＿＿＿＿＿＿＿＿＿＿＿＿＿＿＿＿＿＿＿＿＿＿＿＿＿＿＿＿＿＿＿＿＿＿＿＿＿＿＿＿＿＿＿＿＿＿＿＿＿＿＿＿＿＿＿＿＿＿＿＿＿＿＿＿＿＿＿＿＿＿＿＿＿＿＿＿＿＿＿＿＿＿＿＿＿＿＿＿＿＿＿＿＿＿＿＿＿＿＿＿＿＿＿＿＿＿＿＿＿＿＿＿＿＿＿＿

**Question 9: Finally, we would like to conclude the discussion, but if there is anything you have left unsaid, please let us know.**

＿＿＿＿＿＿＿＿＿＿＿＿＿＿＿＿＿＿＿＿＿＿＿＿＿＿＿＿＿＿＿＿＿＿＿＿＿＿＿＿＿＿＿＿＿＿＿＿＿＿＿＿＿＿＿＿＿＿＿＿＿＿＿＿＿＿＿＿＿＿＿＿＿＿＿＿＿＿＿＿＿＿＿＿＿＿＿＿＿＿＿＿＿＿＿＿＿＿＿＿＿＿＿＿＿＿＿＿＿＿＿＿＿＿＿＿＿＿＿＿

Thank you very much for your time and cooperation today.
