# Supplementary material for: Barriers and facilitative factors in the implementation of workplace health promotion activities in small and medium-sized enterprises: a qualitative study
Source: Implement Sci Commun. 2022 Mar 2;3:23. doi: 10.1186/s43058-022-00268-4 (PMC8889638; doi:10.1186/s43058-022-00268-4)
Supplement: Supplementary file 3 — Additional file 3: Supplementary file 3. List of interventions implemented in the participated SMEs that were not included in the analysis due to unknown evidence at worksite. [file 43058_2022_268_MOESM3_ESM.docx]

**Supplementary file 3.**

List of interventions implemented in the participated SMEs that were not included in the analysis due to unknown evidence at worksite.

|  |  | Number of enterprises implemented |
| --- | --- | --- |
| Diet | |  |
|  | Setting aside one day a month to not eat sweets | 1 |
|  | Encouraging employees to eat breakfast every day | 1 |
| Physical activity | |  |
|  | Club activities | 2 |
| Infection prevention | |  |
|  | Recommendation of hand washing and gargling | 1 |
|  | Set up hand sanitizer | 2 |
| Others | |  |
|  | Mindfulness meditation | 1 |
|  | Daily checks on mental and physical stress, autonomic nervous system balance, and measurement of vascular health via simple equipment | 1 |
