# Supplementary material for: Barriers and facilitative factors in the implementation of workplace health promotion activities in small and medium-sized enterprises: a qualitative study
Source: Implement Sci Commun. 2022 Mar 2;3:23. doi: 10.1186/s43058-022-00268-4 (PMC8889638; doi:10.1186/s43058-022-00268-4)
Supplement: Supplementary file 4 — Additional file 4: Supplementary file 4. Full version of the results. [file 43058_2022_268_MOESM4_ESM.docx]

**Supplementary file 4: Full version of the results**

**Intervention characteristic domain**

***Intervention Source***

In all the enterprises, the interventions were external as the health nurse-in-charge at the JHIA referred them to adopt the “health declaration,” and WHP activities were selected from the common list compiled by the JHIA. Some employer and/or health managers reported that they could implement the WHP activities soon after the WHP certification system were externally referred because they originally had the motivation to promote and enhance employees’ health and this external referral matched their needs.

*"I also wanted to put a blood pressure monitor in the office and (asked the employees to) measure it, so (after I heard about this “health declaration” system) I immediately consulted with the employer of our company, and we then decided that we should get to work on it right away.” (N, health manager, blood pressure)*

***Evidence Strength and Quality***

The employer's perception of the effectiveness of the activities was a strong influencing factor. An ex-smoking employer, who perceived treatment clinics to be effective for smoking cessation from personal experience, adopted a measure to provide a treatment fee subsidy and took a strong leadership position to encourage all the smokers within the enterprise to visit the clinic.

*" (The employer said) let's go a little further and make it this way. (The employer is) a person who quit smoking himself. (Omit) It's a smoking cessation clinic. He thought it would have an outstanding effect. (Omit) It was semi-compulsory, though." (I, health manager, tobacco control)*

***Relative Advantage***

When deciding on a topic for the WHP activity, when the health manager recognized its relative advantages over other topics within NCD prevention, it was more likely to be selected and be proactively implemented. At one enterprise, the health manager selected exercise since it is relevant for all ages and allows everyone to participate in and benefit from it, as compared to other interventions such as those related to smoking or blood pressure.

*"As for anti-smoking measures, (Omit) there were hardly any smokers, so I thought there was no need to do much. There were some people with high blood pressure who were taking medication, but (Omit), it was only people in their 60s, so (Omit), we had to promote something that impact on the whole company (everyone in the company), (Omit), and I felt that exercise promotion might be the best idea, so I decided to promote exercise." (A, health manager, physical activity)*

***Complexities***

Some health managers mentioned that they chose the activities among the list JHIA nurses provided in terms of being easy to start. Public health nurses also mentioned that activities recognized as being easy and less complex to adopt by health managers is one of the key factors that played a role in whether they actually implement the activities afterwards.

**Outer setting domain**

***Patient Needs and Resources***

None of the workplaces followed a direct way to understand employee needs or to elicit feedback, such as administering questionnaires. However, the formal system (e.g., health committees responsible for bringing the employees’ voices to the forefront) or informal way (e.g., feedback shared during daily communication) were used and such employers’ and health managers’ understanding of employee needs facilitated the implementation, especially by increasing the compatibility.

*“We only had one blood pressure monitor. In fact. It took about a minute to measure the blood pressure, and it took a few minutes including taking a record, so there was a traffic jam. They didn't like that, so I negotiated with the company to buy two more blood pressure monitors, and now we have three in total.”  (J, health manager, blood pressure)*

Alternately, fulfilling the needs of employees may inhibit and even draw back the implementation, in the case of tobacco control measures. For example, one enterprise had originally prohibited smoking in the whole workplace area, but this soon became a cause of distress for those who smoked. Subsequently, they consulted with a tobacco company and set up a smoking area outside the premises.

*“I didn't have much knowledge, see, people would say things about stress and whatnot, and I did not even know how stress from not to smoking could be avoided, so I called XX (tobacco company name).” (J, health manager, tobacco control)*

***Cosmopolitanism***

One health manager mentioned the advantage of networking with other companies on program implementation. In the case of company located within an industrial park sharing the health checkup bus, the implementation of health checkup was perceived to be highly advantageous in terms of leading to a collaboration with other organizations in the industrial park.

*"Now, all of the employees in this industrial park gather (to receive health checkups). Until four or five years ago, only our company had done them." (D, health manager, health checkups)*

The health manager of the cooperative union (the enterprise recruited as an interview target, and later treated as complementary data same as interviews of public health nurses) reported that it was effective to make an opportunity for health managers from various companies to meet each other and share their concerns and ideas, as most of them were conducting WHP activities by themselves.

***Peer Pressure***

One health manager reported that she feels pressure from the group companies having a competitive relationship. The health manager at this enterprise had obtained the certification of WHP by the government under “external policy and incentives” and was proud of being a leader in health management when compared to other group companies. This increased her “self-efficacy” and made continuous implementation more likely.

*“After all, both the prefecture and the national government renew the program every year, so it's not like once you get it, it's good forever. So, I am aware that we have to work hard every year. If I don't get Gold this year, I’ll be disappointed.” (I, health manager, tobacco control)*

***External Policy and Incentives***

One health manager reported that legal obligation was a strong incentive to focus on during the implementation of health checkups by increasing the “relative advantage” of its implementation.

*“Last year, a public health nurse (Omit) told us to do our best because it could be considered a violation of the company's obligations under the Industrial Safety and Health Act. I have renewed my mind since last year that I would like to keep this in mind.” (C, health manager, health checkup)*

Additionally, obtaining the certification had an impact not only on those outside the company but also on awareness among the employees of the company. That is, being aware of the company’s commitment to health promotion, and witnessing it daily improved employees' awareness of WHP activities as well.

*"Yeah. This (health declaration plaque) is propped up in the entrance, where the time clock is placed. Here, everyone will see it. It says, 'This is what our company is doing.' This makes a big difference in awareness." (D, health manager, tobacco control)*

**Inner setting domain**

***Networks and Communication***

Within the enterprises, formal networks such as health committees had a role to play in the two-way communication; they were responsible for bringing the employees’ voices to the forefront as well as for communicating health-related information to the employees. Such internal communication facilitated implementation.

*“The Health and Safety Committee members (collect and) represent the workplaces (employees opinions), as I'm sure they are listening various opinions from the employees.” (F, employer, tobacco control and blood pressure)*

Alternatively, relationships of mutual trust between the employer and the employees, and the employer’s sincere concern for the employees and the employees’ desire to respond to the employer’s concern for them, facilitated implementation.

*“Unlike large companies, we work with a small number of people, so we can keep an eye on things to a certain extent. If she (health manager) notices that employees have lost weight or gained weight, she will ask you, "Have you been eating vegetables lately?"” (M, employer, blood pressure)*

***Culture***

In two of the enterprises consisting of fewer than 30 employees, the health managers’ sincere concern for the health of their employees created a family-like culture in the workplace. Such a culture encouraged leaders, managers, and employees to communicate even minor health changes, and promoted the WHP activities (see also “networks and communications”).

**Tension for Change**

Some employers and health managers mentioned two kinds of sense of urgency for change. First, they reported that a sense of urgency from the employer or health manager, especially in small establishments with fewer than 30 employees, arose from the fact that they would be in real trouble if even one of their employees fell ill.

*"Nowadays, in the transportation industry, which is also the case in other industries, even if we engage in recruiting, people just don't come, and from such a point of view, we have to maintain the people we have. If they are hospitalized, we are in trouble.” (M, health manager, blood pressure)*

Second, a sense of urgency resulting from existing employee health issues arose, such as a higher prevalence of smoking within the company or sudden onset of cancer or heart disease among employees.

*“An outsourced driver once had a myocardial infarction while driving a dump truck and broke a traffic light.” (B, health manager, blood pressure)*

***Compatibility***

Many health managers reported that alignment with employees’ business processes promoted the continuation of implementation. For example, a 100% participation in health guidance was achieved among eligible employees by making the time and access less disruptive to their work.

*“(People who are subject to health guidance after the health checkup) are mostly busy with work, so I asked a public health nurse to come early before going out in the morning, and we make time for about 30 minutes from 9:00 am to 9:30 am.” (D, health manager, health checkups)*

Alternately, if the activity was incompatible with employees' work, such as difficulty in coordinating the schedule for implementation, it inhibited implementation. Many of the hospitals limit the health examinations to weekdays and Saturday mornings, which is incompatible with the work schedule of employees in the transportation industry who generally work on all weekdays and holidays.

*"I think it is necessary for people to recognize that there are many different types of businesses in the world. (Omit) It would be easier for us if the hospitals would consider the system for receiving patients.” (M, employer, blood pressure)*

The time required for the activity was reported to be a barrier to its implementation. In one of the enterprises, despite the health and safety consultant recommending that blood pressure be checked regularly at the construction site, the health manager perceived this to be impractical since blood pressure examinations could not be conducted for hundreds of people every morning before work with only one sphygmomanometer; as a result, a sphygmomanometer was brought at the construction site, but the checking of the blood pressure was optional and not routinized.

*"The consultant says that there are some companies that have forced all the employees to measure it (blood pressure) and kept records of it. But in terms of whether or not that will continue, well, we can only do it to the extent that is lasts." (B, health manager, blood pressure)*

***Relative Priority***

Many health managers mentioned that the enterprise’s prioritization of WHP activities was relative to other things as a facilitative factor. Specifically, if health management was a part of the company’s overall management vision, it was easy to obtain the leader's approval and implement health promotion measures immediately.

*"It's going to cost, and we talked to the employer and (health and safety) committee. (Omit) The most important thing was that it would help employees manage their health. So, we got the go-ahead right away." (F, health manager, tobacco control)*

However, one employer mentioned that WHP implementation was a lower priority compared to customer-focused activities or productivity. Such a relatively low priority can be a barrier to implementation, and is likely to be highly dependent on the business conditions of SMEs at any given time.

*"At the end of the day, if you have the money, you can do more and more health promotion, but when it comes to where to cut down the budget to make room for it, well, you can't reduce the lump-sum payment given to employees, and you can't reduce salaries. In those situations, I think it eventually leads to discussions that we have to cut such-and-such areas."　(C, employer, blood pressure)*

***Organizational Incentives and Rewards***

One health manager reported that praising the participants of WHP activities in a meeting promoted the implementation, especially employees’ participation. Praising not only motivates the employees themselves, but also makes all the employees who see them realize that participation in WHP activities is something that the company considers important and a high priority.

*"We often have meetings at our place. When we gather everyone for a meeting, we praise them for those things (health checkups and health examination results)."　(N, health manager, blood pressure and health checkups)*

***Goals and Feedback***

One health manager reported that the employers repeatedly linked the goals and objectives of the WHP activities to the company’s management policies during periodic meetings with employees. This creation of a common understanding of the goals and objectives for WHP activities led to employees’ favorable attitudes toward participation in the WHP activities.

*"(At the regular monthly meeting) We discuss each event in turn. For example, when we have a sports day at the end of this month or next month, we ask (the employer) to tell employees why we are doing it. (Omit) We make sure that the employer explains the purpose of these things and why we’re engaging in this stuff as a company." (A, health manager, physical activity)*

***Learning Climate***

When health managers feel that the employer perceived them as an indispensable and knowledgeable person in the WHP implementation, they proactively examine, plan, and implement the WHP activities. In one enterprise, the health manager, who previously had no knowledge of health management, but was trusted by the employer and assigned this task, proactively implemented the program through trial and error. When the implementation went well, the manager felt affirmed, raising their “self-efficacy”, and the motivation to continue the program, and the implementation of other activities further increased, thereby, creating a virtuous cycle.

*"The representative just told me he wanted to do health management for the employees. It was a great learning experience for me to work on our own." "(When deciding on the WHP activities to adopt) The employer basically gave me permission to select whichever I wanted. (Omit) I didn't ask (my superiors) which one they preferred. We kind of just said, ‘This is the one we'll go with" (A, health manager, physical activity)*

***Leadership Engagement***

There were two ways in which employers engaged in WHP activities—communicating the company's philosophy linked to the WHP to all the employees, and supporting those who are engaging the implementation—both of which were strong drivers of implementation. Direct and repeated communication from the employer at general meetings and other occasions led others within the company to relatively prioritize WHP activities more and, hence, implementation progressed.

*"The current representative of the company believes that the happiness of employees and those close to them will lead to contributions to customers and the local community. (Omit) I think the most important thing is the representative’s way of thinking." (A, health manager, physical activity)*

Similarly, extending support to those in charge of the program, such as allowing them to participate in external trainings related to WHP program implementation during working hours, facilitated implementation.

*"I was told that I can participate in such things (such as seminars on WHPs outside the company) as much as I want because they see it as part of my work."* *(A, health manager, physical activity)*

Multiple public health nurses/nutritionists in the focus groups supported these findings, as they also mentioned that “The employer’s voice is essential,” and “The influence of employers and health care managers is significant in ensuring the sustainability of WHP implementation.”

On the other hand, health managers who were not given enough time or support to implement WHP-related tasks inevitably gave lower priority for WHP implementation. In this enterprise, one year after declaring that they would perform blood pressure control activities, they still had not purchased a blood pressure monitor.

*"I'd like to help where I can (for implementing WHP activities), but I'm so busy with my other duties and I tend to forget." (C, health manager, blood pressure)*

***Access to Knowledge and Information***

As many SMEs did not have existing resources to initiate WHP activities, many health managers reported that access to external knowledge and information, such as participation in study sessions during working hours and support from JAIH health nurses, was necessary to proceed the implementation. This accessibility to information was enhanced by support from the employer and the positive attitudes of health managers.

*“There are many things that we can't ask what we should do, or can't find the answer to even if we do search. In such cases, when we asked the JHIA nutritionist, she said, "Here is a pamphlet on this and that”. If we don't have that kind of information, we may get lost and end up in trouble.” (L, health manager, diet)*

In contrast, when access to such external knowledge and information was difficult, even if the sense of urgency in the health manager increased, it did not lead to the actual implementation. In one enterprise implementing blood pressure management, nothing was implemented after installing blood pressure monitors despite having a sense of urgency to do something more, because they did not know what to do and had poor access to knowledge and information.

*“There are many employees with high blood pressure, so we need to think of something, but I'm not sure what I can do at work.” (B, health, manager; blood pressure)*

**Characteristics of individuals**

***Knowledge and Beliefs About the Intervention***

Some employers and health managers reported that they were clearly aware of having to conduct WHP activities as part of their regular task, rather than as an additional task, as they believed that the health promotion of employees is one of the issues the enterprise should engage in.

*“Employees are the most important. In order to keep employees to work with high motivation for a long time, (spending resources) for their well-being is an investment, not a cost.” (K, employer, tobacco control and health checkups)*

However, some employers or health managers were convinced that health behavior is no good unless each employee's awareness is changed, and it led to the belief that the WHP activities would have a limited effect, as a result of which the actual implementation was limited.

*"It's not good if the person themselves is not aware of what’s going on. (Omit) I try to do things for myself. (omit) I'm diabetic, so I'm trying hard to lower my blood pressure, but until each of us is aware of it, it won't affect us (no matter what those around us say)." (B, health manager, blood pressure)*

***Self-Efficacy***

Some employers reported, or health manager reported as an employer’s perception, that they (employers) entrusted health managers with the task of health promotion and they were able to accomplish it with the help of adequate time and manpower. Then, the managers’ sense of self-efficacy increased, thereby leading to a virtuous cycle and continued implementation in the subsequent years (see “learning climate” and “peer pressure” as well).

***Individual Identification with Organization***

Some health managers described that the employer’s sincere concern for the employees lead the employees' desire to respond to the employer's concern for them, and such relationships of mutual trust between the employer and the employees facilitated implementation.

“The employer is very considerate of us, so we want to respond to him as well.” (I, health manager, tobacco control)

**Process domain**

***Opinion Leaders***

One health manager reported that by identifying an opinion leader and getting her to become an advocate first, he gradually increased the involvement of employees and reduced their resistance to change and new activities.

*"In the beginning, I explained this project to a senior person who has a strong influential power in the company first, because it will definitely be the useful way to spread the activities. (A, health manager, physical activity)*

***Formally Appointed Internal Implementation Leaders***

All enterprises formally appointed a health manager to implement WHP activities as doing so was mandated when they had adopted the “health declaration” certified by the JHIA. Some health managers were strongly motivated and acted as a front-line champion as well. On the other hand, when the employer concurrently served as the health manager, the absence of the appointed health manager who could fill the gap between the employer and employees in terms of their perception of WHP activities, was a barrier for implementation, especially for employee participation in the activities.

***Change Agent***

Most of employers and health managers perceived the public health nurses or nutritionists at JHIA as key members when implementing WHP activities, as they provided useful advice or information about WHP. In addition, they perceived that health lectures by public health nurses are more effective as employees were more receptive to the information coming from them.

*"I frequently read the health bulletins from the JHIA (Omit), and I can see that other establishments are doing such things. I also ask, ‘What are other places doing?’ ‘What do other places do?’ (Omit) I receive some guidance and information from the public health nurses.” (N, health manager, blood pressure)*

***Reflecting and Evaluating***

None of the workplaces conducted quantitative or qualitative evaluations of the progress and quality of the WHP activities by themselves. However, one health manager reported that she conducted a personal reflection using an annual health report by JHIA, which indicated the average scores of the health examination at their enterprise. The health manager set a goal to achieve blood pressure levels below the average of other industries, and was encouraged to implement WHP activities that helped in moving towards the goal.

*"I'm hoping that when I receive it (the office health report) from JHIA again, it’ll be a little bit closer [to these sort of results], better than in (the average of) Prefecture A or than other companies.” (N, health manager, blood pressure)*
