# Supplementary material for: Barriers and facilitative factors in the implementation of workplace health promotion activities in small and medium-sized enterprises: a qualitative study
Source: Implement Sci Commun. 2022 Mar 2;3:23. doi: 10.1186/s43058-022-00268-4 (PMC8889638; doi:10.1186/s43058-022-00268-4)
Supplement: Supplementary file 5 — Additional file 5: Supplementary file 5. Summary of the findings from the focus groups. [file 43058_2022_268_MOESM5_ESM.docx]

**Supplementary file 5: Summary of the findings from the focus groups**

This description consists of a summary of the findings from the focus groups with public health nurses and one enterprise using content analysis based on CFIR constructs.

**INTERVENTION CHARACTERISTICS**

**Complexities**

*Activities recognized as being easy*

If the health manager felt that the activities were easy, they would actually implement them. Activities need to be recognized as being easy for them to select and adopt the same.

**OUTER SETTING**

**Patients' needs and resources**

*Restraint/consideration from the employer’s end (barrier)*

Regarding tobacco control measures, the employers were hesitant to strongly promote tobacco control due to reluctance to deal with employees and business partners who smoke. Due to the difficulty in hiring new personnel and the desire to not lose even one employee, the employers were afraid that employees who smoke will quit if tobacco control was adopted, which was a strong disincentive for them.

**Peer pressure**

*The situation regarding WHP in other enterprises*

Many of the enterprises were interested in health promotion efforts and health examination values implemented by other enterprises in the same industry. JHIA's annual health report proved to be a motivating tool for employers and staff members.

**INNER SETTING**

**Structures**

*Company size*

The smaller the company size (e.g., 10 employees), the easier it is to reach out to employees.

**Relative priority**

*Aligning with the enterprise’s priorities*

Aligning with the enterprise’s priorities was a crucial point. For instance, for many enterprises, appealing to the effects of WHP activities on management and human resources, rather than making health the sole focus would be more effective.

*Addressing the existing health issues within the enterprise*

The program needed to be useful in resolving the existing health issues within the enterprise. If the program could solve the health-related issues employees were facing at the time of implementation, it would be easily understood and supported by the employer and health manager, and implementation would proceed smoothly. Alternately, it could be perceived as an imposition, and implementation would be hindered.

**Network and communications**

*Close communication across the departments in the enterprise*

In many cases, health managers were the administrative staff, and with an absence of close communication between departments, it may have been difficult for WHP activities to penetrate the whole company (e.g., when the office and factory are separated, even if a young health manager tells the factory employees who were in their 50s and 60s about the need for health promotion activities, they may not listen to him/her). Having a system for regular communication between departments was a facilitating factor for implementation.

**Leadership engagement**

*The top-down approach*

The top-down approach was effective especially in companies with fewer than 50 employees as the employer's voice had a strong influential power. It was also effective for employers and executives to talk about the progress of WHP activities during morning meetings for its sustained implementation.

**INDIVIDUAL CHARACTERISTICS**

**Health manager's characteristics**

*Health manager's skills and authority*

The health manager's skills and authority influenced the implementation directly. Although it was not necessarily related to their position, it was important for health managers to have the authority to speak in such a way that employees would listen to them, especially with respect to the continuity of WHP activities.

**PROCESS**

**Champions**

*Involving front-line champions*

Involving front-line champions advanced program implementation. For example, when adopting measures to make the company cafeteria menu healthier, discussing the issue among all stakeholders i.e., not only the employer and general manager, but also the cook, led to the program’s successful adoption and implementation.

*Note*. WHP: Workplace health promotion; CFIR: Consolidated framework for implementation research; JHIA: Japan Health Insurance Association
