# Supplementary material for: Barriers and facilitative factors in the implementation of workplace health promotion activities in small and medium-sized enterprises: a qualitative study
Source: Implement Sci Commun. 2022 Mar 2;3:23. doi: 10.1186/s43058-022-00268-4 (PMC8889638; doi:10.1186/s43058-022-00268-4)
Supplement: Supplementary file 6 — Additional file 6: Supplementary file 6. Barriers and facilitators in workplace health promotion according to topics of activities. [file 43058_2022_268_MOESM6_ESM.docx]

**Supplementary file 4**

**Supplementary table 1: Barriers and facilitators in workplace health promotion according to topics of activities**

|  |  | Smoke-free policies | | | | | | |  | Regular checks of blood pressure | | | | | |  | Diet |  | Physical activity | |  | Health checkups | | | | | |
| --- | --- | --- | --- | --- | --- | --- | --- | --- | --- | --- | --- | --- | --- | --- | --- | --- | --- | --- | --- | --- | --- | --- | --- | --- | --- | --- | --- |
|  |  | Comprehensive bans | | | | Partial bans | | |  | Regular monitoring of blood pressure | | | Only placing sphygmomanometer | | Not yet placing sphygmomanometer |  | Providing healthy menu at lunch time |  | Physical activity programs | |  | 100% implementation rate of health checkups and healthcare advice | | | | | |
| Enterprise | | D | H | I | K | E | F | J |  | J | M | N | B | F | C |  | L |  | A | J |  | C | D | E | G | K | N |
| I. Intervention Characteristics | |  |  |  |  |  |  |  |  |  |  |  |  |  |  |  |  |  |  |  |  |  |  |  |  |  |  |
|  | A. Intervention Source | + |  |  |  |  |  |  |  |  | + | + |  |  |  |  |  |  |  |  |  |  |  |  |  |  |  |
|  | B. Evidence Strength & Quality |  | + | + |  |  |  |  |  |  |  |  |  |  |  |  |  |  |  |  |  |  |  |  |  |  |  |
|  | C. Relative Advantage |  |  |  |  |  |  |  |  |  |  |  | + |  |  |  |  |  | + |  |  |  | + | + |  |  |  |
|  | F. Complexities |  |  |  |  |  |  |  |  |  |  |  |  |  |  |  |  |  | + | + |  |  |  | + |  |  |  |
| II. Outer Setting | |  |  |  |  |  |  |  |  |  |  |  |  |  |  |  |  |  |  |  |  |  |  |  |  |  |  |
|  | A. Needs & Resources of Those Served by the Organization |  | + |  | − |  | + | − |  | + |  |  |  |  |  |  | + |  |  |  |  |  |  |  | + |  |  |
|  | B. Cosmopolitanism |  |  |  | + |  | + |  |  |  |  |  |  |  |  |  |  |  |  |  |  |  | + |  | + |  |  |
|  | C. Peer Pressure |  |  | + |  |  |  |  |  |  |  | + |  |  |  |  |  |  |  |  |  |  |  |  |  |  | + |
|  | D. External Policy & Incentives | + |  | + |  |  | + |  |  |  |  |  |  |  |  |  |  |  |  |  |  | + |  |  |  |  |  |
| III. Inner Setting | |  |  |  |  |  |  |  |  |  |  |  |  |  |  |  |  |  |  |  |  |  |  |  |  |  |  |
|  | B. Networks & Communications |  | + | + |  | + | + | + |  | + | + | + |  | + |  |  | + |  |  | + |  |  |  |  | + |  | + |
|  | C. Culture |  |  |  |  |  |  |  |  |  | + | + |  |  |  |  |  |  |  |  |  |  |  |  | + |  | + |
|  | D. Implementation Climate |  |  |  |  |  |  |  |  |  |  |  |  |  |  |  |  |  |  |  |  |  |  |  | + |  |  |
|  | 1. Tension for Change |  |  | + | + |  | + |  |  | + | + |  | + |  | − |  | + |  | + |  |  |  |  |  |  | + |  |
|  | 2. Compatibility |  |  |  |  | + |  |  |  | + | + | + | − |  |  |  | + |  |  |  |  |  | + | + |  |  | + |
|  | 3. Relative Priority |  | + | + |  |  | + |  |  | + |  | + |  | + | − |  |  |  | + |  |  |  |  |  |  | − | + |
|  | 4. Organizational Incentives & Rewards |  |  | + |  |  |  |  |  |  |  | + |  |  |  |  |  |  |  |  |  |  |  |  |  |  |  |
|  | 5. Goals & Feedback |  |  | + |  |  |  |  |  |  |  |  |  |  |  |  |  |  | + |  |  |  |  |  |  |  |  |
|  | 6. Learning Climate |  |  |  |  |  |  |  |  |  |  |  |  |  |  |  | + |  | + |  |  |  |  |  |  |  |  |
|  | E. Readiness for Implementation |  |  |  |  |  |  |  |  |  |  |  |  |  |  |  |  |  | + |  |  |  |  |  |  |  |  |
|  | 1. Leadership Engagement | + |  | + |  |  | + |  |  | + |  | + |  | + | − |  | + |  | + |  |  |  | + | + |  |  | + |
|  | 3. Access to Knowledge & Information |  |  | + | + |  |  |  |  | + | + | + | − |  | − |  | + |  | + |  |  |  |  |  | + | + | + |
| IV. Characteristics of Individuals | |  |  |  |  |  |  |  |  |  |  |  |  |  |  |  |  |  |  |  |  |  |  |  |  |  |  |
|  | A. Knowledge & Beliefs about the Innovation |  |  | + | + |  | + | − |  |  | + | + | − | + |  |  | + |  | + |  |  |  |  |  | + | + | + |
|  | B. Self-Efficacy |  |  | + |  |  |  |  |  |  |  |  |  |  |  |  |  |  | + |  |  |  |  |  | + |  |  |
|  | D. Individual Identification with  Organization |  | + | + |  |  |  |  |  |  |  |  |  |  |  |  |  |  |  |  |  |  |  |  |  |  |  |
| V. Process | |  |  |  |  |  |  |  |  |  |  |  |  |  |  |  |  |  |  |  |  |  |  |  |  |  |  |
|  | 1.Opinion leaders |  |  |  |  |  |  |  |  |  |  |  |  |  |  |  |  |  | + |  |  |  |  |  |  |  |  |
|  | 2. Formally Appointed Internal Implementation Leaders | + | + | + | − | + | + | + |  | + | + | + | + | + | + |  | + |  | + | + |  | + | + | + | + | − | + |
|  | 4. External Change Agents |  | + | + | + |  |  |  |  |  |  | + |  |  |  |  |  |  |  |  |  | + |  |  | + | + | + |
|  | D. Reflecting & Evaluating |  |  |  |  |  |  |  |  |  |  | + |  |  |  |  | + |  | + |  |  |  |  |  | + |  |  |
